# Dispersal, niche, and isolation processes jointly explain species turnover patterns of nonvolant small mammals in a large mountainous region of China

**DOI:** 10.1002/ece3.1962

**Published:** 2016-01-18

**Authors:** Zhixin Wen, Qing Quan, Yuanbao Du, Lin Xia, Deyan Ge, Qisen Yang

**Affiliations:** ^1^ Key Laboratory of Zoological Systematics and EvolutionInstitute of ZoologyChinese Academy of Sciences1 Beichen West RoadBeijing100101China; ^2^ University of Chinese Academy of SciencesYuquan RoadBeijing100049China

**Keywords:** Dispersal processes, distance decay, geographic isolation, halving distance, species turnover, the Hengduan Mountains

## Abstract

Understanding the mechanisms that govern the spatial patterns of species turnover (beta diversity) has been one of the fundamental issues in biogeography. Species turnover is generally recognized as strong in mountainous regions, but the way in which different processes (dispersal, niche, and isolation) have shaped the spatial turnover patterns in mountainous regions remains largely unexplored. Here, we explore the directional and elevational patterns of species turnover for nonvolant small mammals in the Hengduan Mountains of southwest China and distinguish the relative roles of geographic distance, environmental distance, and geographic isolation on the patterns. The spatial turnover was assessed using the halving distance (km), which was the geographic distance that halved the similarity (Jaccard similarity) from its initial value. The halving distance was calculated for the linear, logarithmic, and exponential regression models between Jaccard similarity and geographic distance. We found that the east–west turnover is generally faster than the south–north turnover for high‐latitudinal regions in the Hengduan Mountains and that this pattern corresponds to the geographic structure of the major mountain ranges and rivers that mainly extend in a south–north direction. There is an increasing trend of turnover toward the higher‐elevation zones. Most of the variation in the Jaccard similarity could be explained by the pure effect of geographic distance and the joint effects of geographic distance, environmental distance, and average elevation difference. Our study indicates that dispersal, niche, and isolation processes are all important determinants of the spatial turnover patterns of nonvolant small mammals in the Hengduan Mountains. The spatial configuration of the landscape and geographic isolation can strongly influence the rate of species turnover in mountainous regions at multiple spatial scales.

## Introduction

One central goal in biogeography is to understand the patterns and underlying causes of the variation in species diversity at multiple spatial scales (MacArthur 1972). Species diversity has three scale‐dependent components, which are known as alpha, beta, and gamma diversity (Whittaker 1960). Beta diversity (i.e., species turnover) has been one of the core areas in ecological research over the past decade, but species turnover patterns and their underlying processes at different spatial scales are still not fully explored (Veech and Crist 2007; Anderson et al. 2011). The most commonly reported pattern in species turnover is the decay of community similarity with increasing geographic distance, a phenomenon that has been frequently explained by two mechanisms: dispersal limitation and niche limitation (Soininen et al. 2007; Wen et al. 2014). The idea of dispersal limitation assumes that species distributions are entirely governed by their abilities to disperse and the spatial configuration of the landscape. For the same group of taxa, the higher decay rates of similarity with distance would mostly occur in the landscapes that contain strong dispersal barriers rather than in topographically homogeneous regions; under the same level of topographic heterogeneity, community similarity would decrease more abruptly with distance for less vagile organisms than for more vagile ones (Nekola and White 1999; Hubbell 2001). Under the idea of niche (ecological niche: environmental factors that are necessary and sufficient to allow a species to survive and reproduce, James et al. 1984) limitation, the distance decay in similarity is purely caused by the increasing difference in environmental characteristics with distance, with the rate at which the community composition changes being faster in the region with larger environmental gradients (Tilman 1982). In reality, dispersal and niche processes are not mutually exclusive; in most cases, they work together in driving species turnover, although their relative importance may vary considerably across different taxa and geographic regions (Buckley and Jetz 2008; Astorga et al. 2012; Wang et al. 2013). Additionally, because many environmental factors are often spatially structured (Peres‐Neto et al. 2012), it remains a challenge to disentangle the relative contributions of dispersal and niche processes and thereby explain patterns of species turnover at broad geographic scales, particularly in studies that use a large resolution and a large spatial extent (Heino and Alahuhta 2015).

In addition to dispersal and niche processes, geographic isolation and historical processes (e.g., speciation, extinction, and dispersal‐recolonization dynamics) have long been recognized as major drivers of geographic patterns of species turnover, although the assessment of their relative roles is generally lacking (Svenning et al. 2011). The impacts of geographic isolation and historical processes on species turnover are especially pronounced in regions where geologic and climatic events occur constantly. Among the various types of geologic changes, the uplift of mountain ranges is an important driver of species differentiation, primarily by promoting allopatric speciation and endemism as a result of geographic isolation. Previous studies have clearly demonstrated the effects of orogeny‐driven isolation on species turnover for vertebrates (Melo et al. 2009) and plants (Qian et al. 2013). Climatic oscillations, and especially those that occurred in the Quaternary, were a key historical factor in shaping the distributions and turnover of contemporary species, and their effects can be observed in many parts of the world (Leprieur et al. 2011). Arguably, there is more than one way in which Quaternary climatic oscillations may have affected the pattern. For example, in glacial periods, species differentiation among sites could emerge through the extinction of species in high‐elevation and high‐latitude areas or through the persistence and evolution of species in low elevation and latitude refugia (Svenning et al. 2011; Dobrovolski et al. 2012). During the interglacial and postglacial periods, recolonization from refugia may become a major mechanism for species turnover, as found in North American mammals (Qian et al. 2009) and European longhorn beetles (Baselga 2010). Because of the importance of geographic isolation and historical processes, it is essential to include an evaluation of their effects on spatial turnover patterns, although it is often difficult to disentangle the roles of present‐day and historical factors due to co‐varying climatic conditions (Heino and Alahuhta 2015).

Previous species turnover studies have mainly focused on the organisms in flatland landscapes (e.g., Duivenvoorden et al. 2002; Tuomisto et al. 2003), but very few have specifically investigated montane biota (but see Mena and Vázquez‐Domínguez 2005). In contrast to plains, mountainous regions are generally characterized by rapid elevation changes and spatial variation in topography, climate, and vegetation. These conditions may be related to the high species turnover explained by dispersal limitation and high environmental heterogeneity (Melo et al. 2009; Qian et al. 2013). Moreover, most mountainous regions of the world have experienced dramatic geologic changes, which triggered a series of isolation events that favor species diversity and endemism (Leprieur et al. 2011). Consequently, mountainous regions are ideal models to study the effects of different processes (dispersal, niche, and isolation) on the spatial patterns of species turnover.

In this study, we examined the spatial turnover patterns of nonvolant small mammals in the Hengduan Mountains of southwest China, a typical mountainous region and one of the world's 25 biodiversity hot spots (Myers et al. 2000). The nonvolant small mammals considered in our study include the orders Erinaceomorpha, Soricomorpha, Scandentia, Lagomorpha, and Rodentia (Wu et al. 2013). There are several reasons that make the Hengduan Mountains a suitable region to identify the roles of different processes in structuring large‐scale patterns of species turnover. First, the Hengduan Mountains spans a broad spatial area that is characterized by many major mountains and rivers that extend mainly in a south–north direction. Thus, it provides an opportunity to explore the influence of dispersal processes by examining the directional patterns of species turnover. Second, the region contains millions of extensive elevational gradients in which numerous habitats occur (Zhang et al. 1997a), thus making it an interesting region to study the effects of niche processes on community similarity. Third, strong geologic and climatic changes since the Pleistocene have resulted in complex evolutionary dynamics for small mammals here (Shi et al. 1998; Chen et al. 2010). Studies of species turnover can illuminate our understanding of how geographic isolation and historical processes such as speciation, dispersal, and recolonization have created regional species diversity and differentiation between sites or regions. Additionally, if geographic isolation indeed plays a role, it is expected that there will be different turnover rates in different elevation zones of the Hengduan Mountains because isolation typically increases with elevation.

This study aimed to (1) distinguish the relative effects of geographic distance, environmental distance, and geographic isolation on the species turnover patterns of nonvolant small mammals in the Hengduan Mountains; (2) explore whether the spatial configuration of the landscape controls the spatial turnover in mountainous regions; and (3) determine whether the increasing geographic isolation with elevation would cause any elevational patterns of species turnover in mountainous regions.

## Materials and Methods

### Study area

**Figure 1:**
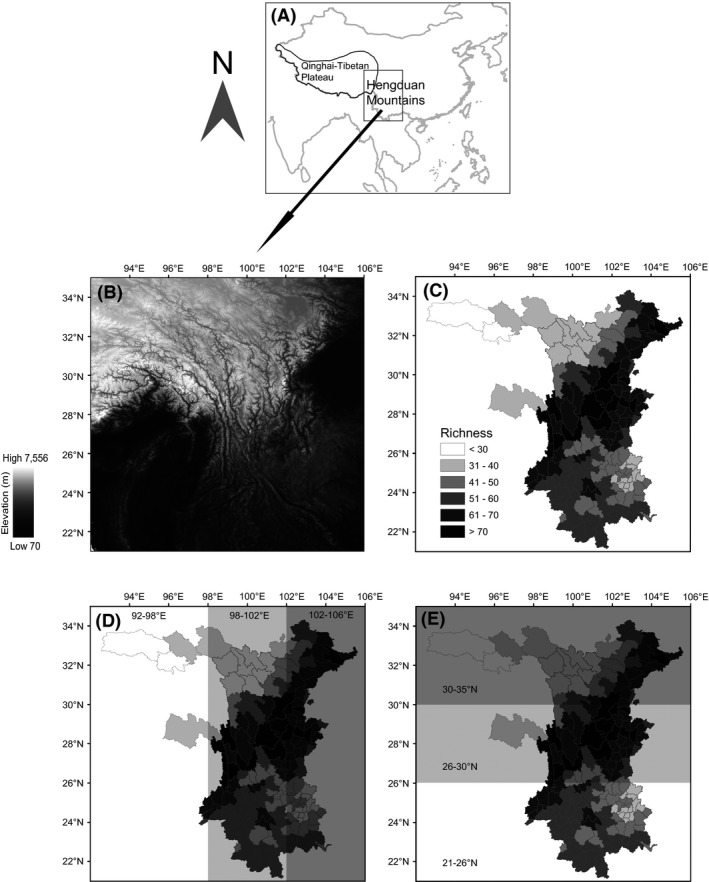
Maps showing the (A) location, (B) species richness of nonvolant small mammals in the 164 counties, (C) topography, (D) three longitudinal zones, and (E) three latitudinal zones of the Hengduan Mountains.

The Hengduan Mountains (21°–35°N, 92°–106°E) lies to the southeast of the Qinghai–Tibetan Plateau (Fig. 1A) and covers a total area of approximately 740,000 km^2^ (data from the National Fundamental Geographic Information System of China, http://nfgis.nsdi.gov.cn/nfgis/). This region includes the southern part of Qinghai Province, the northeastern part of the Tibet Autonomous Region, the western portion of Sichuan Province, and the western part of Yunnan Province. The Hengduan Mountains has an enormous elevation range of 7480 m (76 to 7556 m), with the terrain generally sloping downward from northwest to southeast (Fig. 1B). This geographic feature leads to a wide range of habitat types in this region, which range from the lowland tropical rainforest in southwestern Yunnan to the alpine permafrost in the mountainous region of central Sichuan.

### Species distribution data

We compiled a comprehensive dataset containing the city‐/county‐level (cities are regarded as counties hereafter) distribution information of nonvolant small mammals. Because county‐level data are the main form in which the distribution information of taxa [collected by the Chinese Academy of Sciences (CAS), colleges and universities, local research institutions, and epidemic prevention station] is documented in the Hengduan Mountains, we used the county level as the spatial resolution of this study. Chinese administrative units (e.g., county and province) have frequently been used as data points in large‐scale beta diversity analyses (Chen et al. 2011; Wang et al. 2012). Although we note this may raise potential area issues because area varies among counties, the effect of the difference in county size on the compositional differences in the Hengduan Mountains is low (see Results). Additionally, using gridded data derived from the county‐level distribution data may overestimate the grid species richness because some counties with large areas would be divided into parts belonging to more than one grid cell after rasterization. For species that have a narrow distribution in a large county, the presence could be recorded in the cells in which it is actually absent. The county‐level distribution data were extracted from museum records (Mammal Collections of Institution of Zoology, CAS; Zoological Museum of China West Normal University), a scientific database (Animal Resource Database of Southwest China of Kunming Institution of Zoology, CAS, http://www.swanimal.csdb.cn/swcountyvertb/), books and faunistic atlases (Zhang et al. 1997a; Wang 1998, 2003; Wang and Hu 1999; Zhang 1999; Smith and Xie 2008), and research papers and survey reports of nature reserves. Data from seven counties in the Tibet Autonomous Region are lacking, and these counties were thus excluded from the dataset to reduce analytical biases. We also consulted two taxonomists to avoid dubious records. As a result, the final dataset contained information on 191 species from five orders, 14 families, and 80 genera, according to the system of Wilson and Reeder (2005a,b) (Appendices S1 and S2). The dataset covers 164 counties, which account for 95.9% of all counties and 90.1% of the land area of the Hengduan Mountains (county area ranges from 455 to 34,840 km^2^, and the mean area is 4060 km^2^). The species richness of nonvolant small mammals ranges from 17 to 87 in each county, and the average richness is 55.3 (Fig. 1C).

### Environmental distance

To quantify the effects of environmental distance on species turnover, we measured nine environmental variables in each county that have been demonstrated to strongly affect mammalian diversity at different scales (Currie 1991; Qian et al. 2009) and vertebrate diversity in China (Chen et al. 2011). The variables were mean annual temperature (MAT, °C), mean temperature of the warmest month (MWT, °C), mean temperature of the coldest month (MCT, °C), temperature seasonality (TS = MWT − MCT, °C), mean annual precipitation (MAP, mm), precipitation seasonality (precipitation difference between the wettest and driest month, PS, mm), mean annual potential evapotranspiration (PET, mm), annual normalized difference vegetation index (NDVI), and number of habitat types (NHT; see Appendix S3, Table S1). These parameters encompass environmental information on various aspects of a region, including the ambient energy (MAT, PET, and NDVI), water availability (MAP), and climatic seasonality (TS and PS). The data for MAT, MWT, MCT, MAP, and PS were obtained from the WorldClim Dataset (version 1.4, http://www.worldclim.org/) (Hijmans et al. 2005) at a spatial resolution of 30 arc‐seconds, and the PET data were from the CGIAR‐CSI Global PET Database (1 km^2^, http://www.cgiar-csi.org/) (Zorner et al. 2008). We obtained the NDVI data from the dataset (1 km^2^) at http://www.data.ac.cn/. Following previous research (Qian and Ricklefs 2007), these variables were first subjected to a principal component analysis to reduce the colinearity among the variables based on their correlation matrix. The first four principal components (PC1, PC2, PC3, and PC4) accounted for 94.9% of the total variance and therefore captured most of the information in the raw data (Appendix S3, Table S2). We then calculated the absolute difference on each PC axis between each pair of counties. The environmental distance between pairwise counties, as a single variable, was calculated as the Euclidean distance in the four‐dimensional space of the four extracted PC axes.

### Geographic isolation and environmental heterogeneity

Geographic isolation can create differentiation in species composition among sites by facilitating allopatric speciation. Elevation is an appropriate surrogate of the extent to which a site is isolated from its surroundings, with increasing isolation toward higher elevations. Accordingly, we calculated the differences in the average elevation between counties to test the effects of geographic isolation on species turnover (Leprieur et al. 2011). Environmental heterogeneity, which represents the variability in abiotic conditions within a regional unit, can also influence species turnover (Astorga et al. 2014; Heino et al. 2015). With dispersal and historical factors being consistent, the species turnover between two localities in the same ecoregion tends to decrease with the within‐locality heterogeneity due to the potential for more shared species that are derived from similar niche processes (Qian and Ricklefs 2012). Previous studies on beta diversity have shown that the elevation range is a good proxy variable for environmental heterogeneity (Veech and Crist 2007), so the differences in the elevation range between counties were calculated to evaluate the importance of environmental heterogeneity in affecting spatial patterns. Elevation data were downloaded from the CGIAR‐CSI SRTM 90‐m Digital Elevation Data System (http://srtm.csi.cgiar.org/).

### Spatial species turnover

We employed the Jaccard similarity index (Jaccard 1912) to examine species turnover between each pair of counties, one of the most commonly used similarity indices based on the presence–absence data. The Jaccard index is defined as *J* = *a* / (*a* + *b* + *c*), where *a* is the number of species occurring at both sites and *b* and *c* are the numbers of species that occur at only one site or the other. Values of the Jaccard index range from 0 to 1, which represents a continuum from no species similarity to complete species similarity between the sites. We quantified the spatial species turnover in the Hengduan Mountains using the having distance (km), which was the geographic distance that halved the similarity from its initial value, with a smaller halving distance indicating a faster turnover. Halving distance can be calculated for any type of regression between similarity and distance and is a highly suitable measure for comparative purposes (Soininen et al. 2007). In this study, the relationship between Jaccard similarity and geographic distance was well approximated by the linear, logarithmic, and exponential models in each geographic region (Tables 1 and 2), and we therefore calculated the halving distance of each regression model for comparison. The geographic distances between pairwise counties (km, distance between county centroids) were measured on the World Geodetic System 1984 using the software PAST (version 3.06, http://folk.uio.no/ohammer/past/) (Hammer et al. 2001). To compare the species turnover in the two different directions of the Hengduan Mountains, we divided the region into three longitudinal (92°–98°E: seven counties; 98°–102°E: 85 counties; and 102°–106°E: 72 counties) and three latitudinal (21°–26°N: 81 counties; 26°–30°N: 51 counties; and 30°–35°N: 32 counties) zones (Figs. 1D–E), with the counties grouped by the centroids. Because seven counties in the 92°–98°E zone were omitted due to data deficiency and only several discontinuous counties were remaining, we excluded this zone from further analysis. The spatial species turnover of small mammals in the other five longitudinal/latitudinal zones was assessed using the halving distances of the linear, logarithmic, and exponential models. Furthermore, we compared the spatial turnover in the different elevation zones of the Hengduan Mountains to test whether species turnover is affected by geographic isolation. The counties were subsumed into four elevation zones (<2000 m: 76 counties; 2000–3000 m: 50 counties; 3000–4000 m: 22 counties; and >4000 m: 16 counties) according to their average elevations.

**Table 1:** Halving distance (HD, the geographic distance that halves the similarity from its initial value) (km) in the entire area of the Hengduan Mountains and in the three latitudinal zones, two longitudinal zones, and four elevation zones of the region. The halving distance was calculated for the linear, logarithmic, and exponential regression models between Jaccard similarity of nonvolant small mammals and geographic distance (km) in each geographic region, and each regression was performed with 1000 permutations to determine the statistical significance (*P‐*values, all <0.001)

Geographic region	Initial similarity	*n*	Linear		Logarithmic		Exponential	
			*R* ^2^	HD	*R* ^2^	HD	*R* ^2^	HD
Entire Hengduan Mountains	1	13,366	0.781	402	0.770	325	0.725	348
East–west direction								
21°–26°N zone	1	3240	0.548	464	0.626	557	0.550	488
26°–30°N zone	1	1275	0.645	357	0.664	356	0.622	328
30°–35°N zone	0.967	496	0.740	313	0.779	253	0.814	262
South–north direction								
98°–102°E zone	0.968	3570	0.835	398	0.814	315	0.871	352
102°–106°E zone	1	2556	0.828	418	0.885	312	0.886	365
Elevation zone								
<2000 m	1	2850	0.760	497	0.740	433	0.818	434
2000–3000 m	1	1225	0.666	427	0.733	400	0.698	406
3000–4000 m	0.892	231	0.606	456	0.615	413	0.604	413
>4000 m	0.854	120	0.757	354	0.758	311	0.775	317

**Table 2:** Regression parameters for the relationships between Jaccard similarity of nonvolant small mammals and geographic distance (km) in the entire area of the Hengduan Mountains and in the three latitudinal zones, two longitudinal zones, and four elevation zones of the region. The relationship was approximated by the linear (*y* = *ax* + *b*), logarithmic (*y* = *a*ln*x* + *b*), and exponential (*y* = *be* ^*ax*^) models in each geographic region

Geographic region	Linear		Logarithmic		Exponential	
	*a*	*b*	*a*	*b*	*a*	*b*
Entire Hengduan Mountains	−0.00063	0.7530	−0.2490	1.9401	−0.0019	0.9689
East–west direction						
21°–26°N zone	−0.00066	0.8061	−0.1477	1.4339	−0.0010	0.8147
26°–30°N zone	−0.00087	0.8106	−0.1856	1.5902	−0.0016	0.8456
30°–35°N zone	−0.00078	0.7271	−0.2414	1.8191	−0.0022	0.8610
South–north direction						
98°–102°E zone	−0.00074	0.7782	−0.2638	2.0018	−0.0020	0.9789
102°–106°E zone	−0.00063	0.7634	−0.2188	1.7568	−0.0014	0.8335
Elevation zone						
<2000 m	−0.00057	0.7833	−0.1870	1.6353	−0.0012	0.8421
2000–3000 m	−0.00067	0.7860	−0.1792	1.5738	−0.0012	0.8140
3000–4000 m	−0.00061	0.7241	−0.1752	1.5011	−0.0013	0.7626
>4000 m	−0.00092	0.7528	−0.2440	1.8272	−0.0023	0.8854

Because each county was used for multiple comparisons, the data points of the similarity and the geographic distance in the regressions were not independent. Nonindependence not only has the effect of inflating the degrees of freedom and the assessments of statistical significance, but it also runs the risk of elevating the rates of types I and II error. Consequently, each regression analysis was performed with 1000 permutations in the software Permute! (version 3.4, http://www.bio.umontreal.ca/casgrain/en/labo/permute/index.html) (Casgrain 2001) to determine the statistical significance of the similarity–distance relationship, which has been proved as a robust approach in previous studies (Qian and Ricklefs 2007).

### Species turnover and explanatory factors

To assess the correlations between species turnover and all of the potential explanatory variables, simple and partial Mantel tests were carried out between the Jaccard similarity matrix and the four explanatory matrices (geographic distance, environmental distance, and differences in county average elevation and elevation range) for the entire area of the Hengduan Mountains and for each longitudinal/latitudinal zone, using the ‘mantel’ function of the R package ‘ecodist’ (Goslee and Urban 2007). Next, we applied a variation partitioning method (Borcard et al. 1992; Legendre and Legendre 2012) to distinguish the pure and joint effects of geographic distance, environmental distance, and average elevation difference on species turnover in each region. A series of multiple regressions of the similarity matrix were computed separately on each of the three matrices, and on their different combinations using the ‘MRM’ function of the package ‘ecodist.’ The coefficients of determination (*R* ^2^) were used to partition the variation in species turnover into seven fractions: that explained by each single variable and that by the interactions of multiple variables. Finally, we added the elevation range difference matrix into the regression model. This allowed us to evaluate how much more variance in the species turnover was due to the differences in environmental heterogeneity between sites. We also assessed the amount of the variation additionally explained by difference in county area to determine its effect on the turnover patterns. The Mantel tests and regression analyses were performed with 1000 permutations (see R codes in Appendix S4) in the R programming environment (version 3.0, http://www.r-project.org/).

## Results

### Directional patterns of species turnover

**Figure 2:**
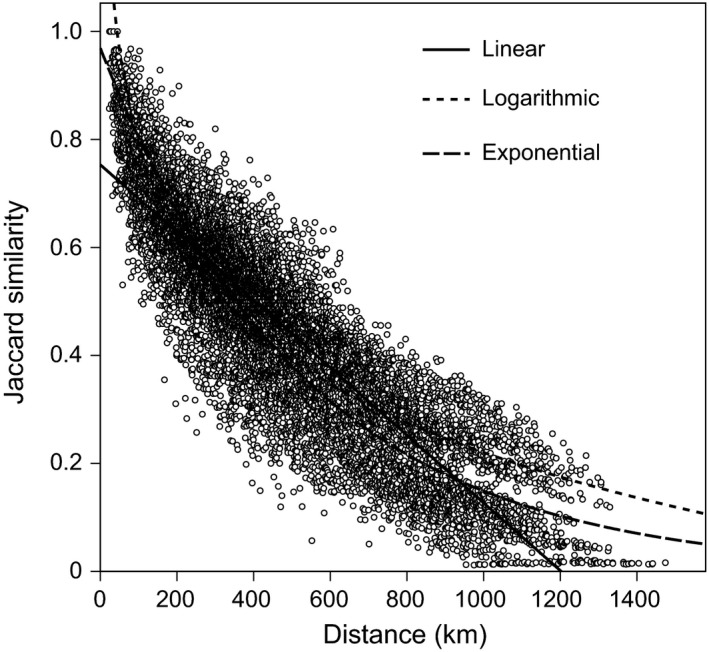
Relationship between the Jaccard similarity of nonvolant small mammals and geographic distance (km) for all pairwise counties (*n *=* *13,366) in the entire area of the Hengduan Mountains. The lines fitted indicate the linear, logarithmic, and exponential regressions, and all the regressions were significant at *P *<* *0.001.

**Figure 3:**
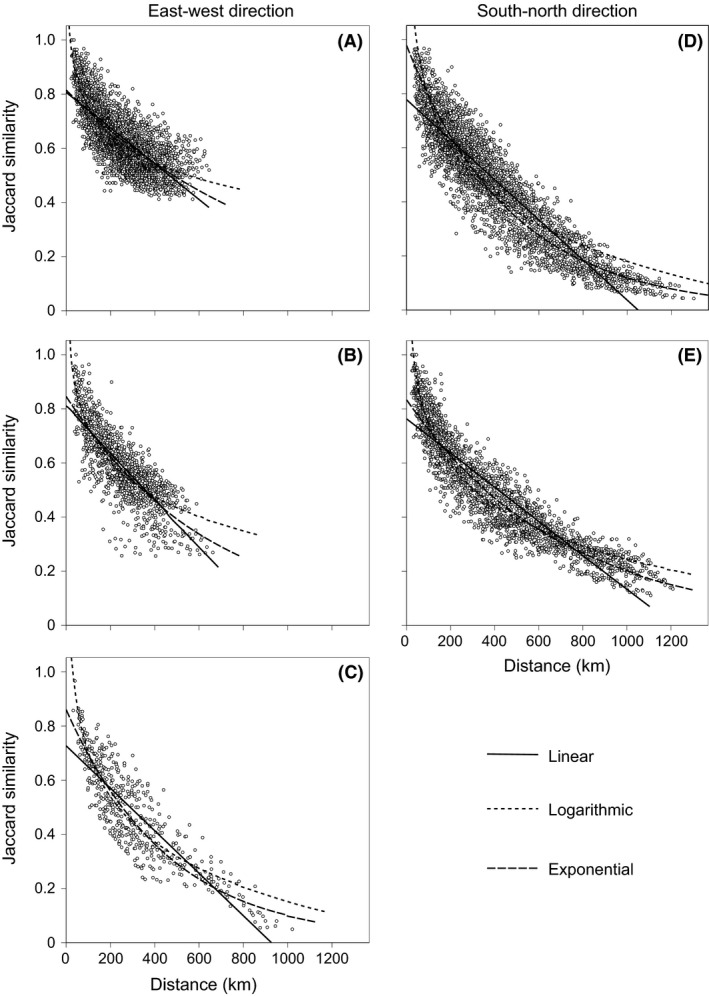
Relationship between the Jaccard similarity of nonvolant small mammals and geographic distance (km) for pairwise counties in the (A) 21°–26°N zone (*n *=* *3240), (B) 26°–30°N zone (*n *=* *1275), (C) 30°–35°N zone (*n *=* *496), (D) 98°–102°E zone (*n *=* *3570), and (E) 102°–106°E zone (*n *=* *2556) of the Hengduan Mountains. For each longitudinal/latitudinal zone, the lines fitted indicate the linear, logarithmic, and exponential regressions, and all the regressions were significant at *P *<* *0.001.

For the 13,366 total pairs of counties in the Hengduan Mountains, the average value of the Jaccard similarity for nonvolant small mammals was 0.45 ± 0.002 (mean ± SE) and ranged from 0.012 to 1. The species similarity between counties decreased significantly with geographic distance, as indicated by the linear, logarithmic, and exponential similarity–distance regression models (all *P *<* *0.001, Fig. 2). The relationship between similarity and geographic distance in each regression model explored was significant (*P *<* *0.001) within all five longitudinal/latitudinal zones (Fig. 3). For both the linear and exponential models, the comparison of halving distances showed that the species turnover in the 26°–30°N and 30°–35°N zones was faster than the turnover in the 98°–102°E and 102°–106°E zones, whereas the turnover in the 21°–26°N zone was the slowest. For the logarithmic model, although the fastest turnover was also observed in the 30°–35°N zone, the halving distance in the 26°–30°N zone was larger than that in the 98°–102°E and 102°–106°E zones (Table 1). Species turnover of nonvolant small mammals is generally faster in the east–west direction than in the south–north direction for high‐latitudinal regions.

### Elevational patterns of species turnover

**Figure 4:**
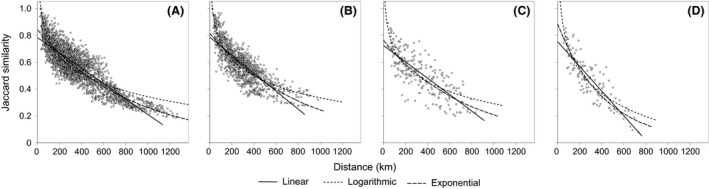
Relationship between the Jaccard similarity of nonvolant small mammals and geographic distance (km) for pairwise counties in the (A) <2000‐m elevation zone (*n *=* *2850), (B) 2000‐ to 3000‐m elevation zone (*n *=* *1225), (C) 3000‐ to 4000‐m elevation zone (*n *=* *231), and (D) >4000‐m elevation zone (*n *=* *120) of the Hengduan Mountains. For each elevation zone, the lines fitted indicate the linear, logarithmic, and exponential regressions, and all the regressions were significant at *P *<* *0.001.

According to all three regression models, the Jaccard similarity between counties decreased significantly with geographic distance in all four elevation zones (*P *<* *0.001, Fig. 4). The spatial turnover generally increases with elevation in the Hengduan Mountains, and this result could be obtained by comparing the halving distances of the linear, logarithmic, and exponential models among elevation zones (Table 1). We also found the same elevational pattern of species turnover measured by the Simpson similarity index which is independent of species richness (see Appendix S5, Table S1 and Figure S1).

### Correlates of species turnover

For the entire area of the Hengduan Mountains, simple Mantel tests indicated that the Jaccard similarity of nonvolant small mammals was negatively correlated with geographic distance, environmental distance, and differences in county average elevation and elevation range (*n *=* *13,366, all *P *<* *0.001). Based on the ranking of correlation coefficients, the similarity showed the highest correlation with geographic distance, and the weakest correlation was observed between the similarity and difference in elevation range (Table 3). According to the partial Mantel tests, the pure effects of all explanatory factors on the similarity were significant, except for difference in elevation range (Appendix S6, Table S1). In all of the longitudinal/latitudinal zones, significant correlations (*P *<* *0.05) between the similarity and all of the explanatory factors were found in the simple Mantel tests (Table 3). However, the correlations of the similarity with several factors became nonsignificant after controlling for the effects of other factors (difference in elevation range in the 21°–26°N, 26°–30°N, 30°–35°N, and 98°–102°E zones; environmental distance in the 26°–30°N zone; difference in average elevation in the 30°–35°N zone; see Appendix S6, Tables S2–S6).

**Table 3:** Results of the simple Mantel tests examining the correlations between Jaccard similarity of nonvolant small mammals and the four explanatory factors (geographic distance, environmental distance, difference in average elevation, and difference in elevation range) in the entire area of the Hengduan Mountains and in the five longitudinal/latitudinal zones of the region. The correlation coefficient and *P‐*value of each test were assessed based on 1000 permutations

Geographic region	Geographic distance (km)	Environment distance	Difference in average difference (m)	Difference in elevation range (m)
Entire Hengduan Mountains	−0.88^a^	−0.61^a^	−0.72^a^	−0.27^a^
East–west direction				
21°–26°N zone	−0.74^a^	−0.47^a^	−0.55^a^	−0.19^a^
26°–30°N zone	−0.80^a^	−0.36^a^	−0.40^a^	−0.23^b^
30°–35°N zone	−0.86^a^	−0.59^a^	−0.44^a^	−0.37^a^
South–north direction				
98°–102°E zone	−0.91^a^	−0.64^a^	−0.84^a^	−0.25^a^
102°–106°E zone	−0.91^a^	−0.64^a^	−0.48^a^	−0.44^a^

^a^ *P* < 0.001; ^b^ *P* < 0.05.

### Relative effects of different factors

**Figure 5:**
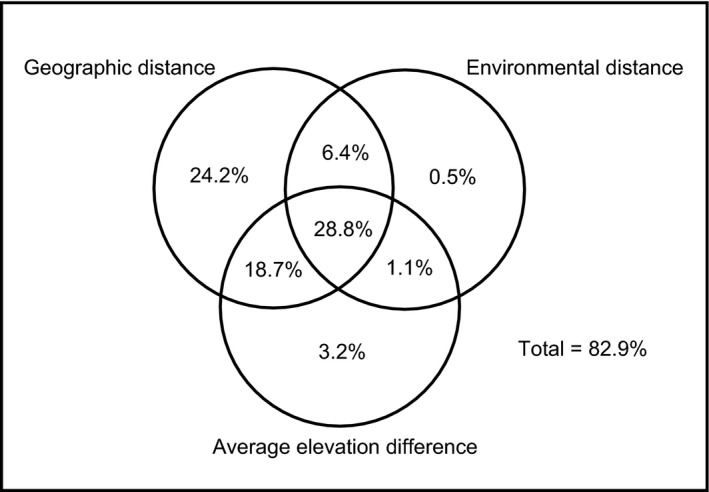
Diagram showing the proportions of the variation in the Jaccard similarity of nonvolant small mammals explained by the pure and joint effects of geographic distance, environmental distance, and average elevation difference in the entire area of the Hengduan Mountains. The coefficients of determination (*R* ^2^) for the multiple regressions that form the basis of variation partitioning are provided in Appendix S7, Table S1.

**Figure 6:**
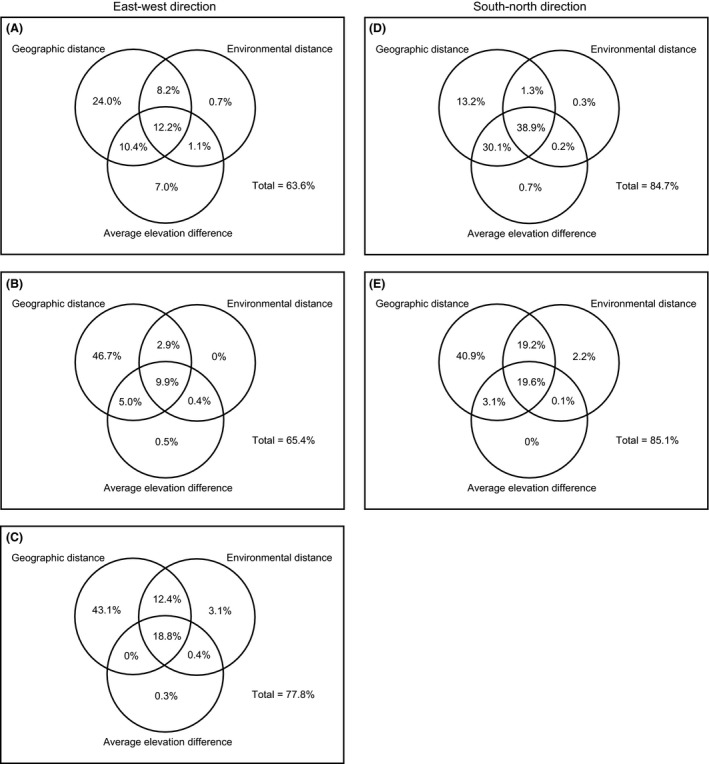
Diagrams showing the proportions of the variation in the Jaccard similarity of nonvolant small mammals explained by the pure and joint effects of geographic distance, environmental distance, and average elevation difference in the (A) 21°–26°N zone, (B) 26°–30°N zone, (C) 30°–35°N zone, (D) 98°–102°E zone, and (E) 102°–106°E zone of the Hengduan Mountains. The coefficients of determination (*R* ^2^) for the multiple regressions that form the basis of variation partitioning are provided in Appendix S7, Table S1.

For the entire area of the Hengduan Mountains, we regressed the Jaccard similarity between pairwise counties against geographic distance, environmental distance, and average elevation difference. The portion of variation in the similarity that can be explained by the three factors was 82.9%, with the second strongest explanatory power derived from geographic distance alone (24.2%). In contrast, the pure effects of environmental distance and average elevation difference were low, independently explaining 0.5% and 3.2% of the variation in the similarity, respectively. The joint effect of the three explanatory factors had a considerable contribution in explaining the turnover (28.8%), as did that of geographic distance and average elevation difference (18.7%, Fig. 5). The total explained variation in the similarity in the east–west direction was smaller, increasing from 63.6% to 77.8% toward the higher latitudes. In each latitudinal zone, most of the explanatory power was purely from geographic distance. However, the pure effects of environmental distance and average elevation difference were weak, except in the 21°–26°N zone (Figs. 6A–C). The total explained variation in the similarity was higher in the south–north direction (84.7% and 85.1%). In the 98°–102°E zone, the largest proportion of the variation was explained by the joint effect of the three explanatory factors (38.9%), and the second strongest explanatory power was from the joint effect of geographic distance and average elevation difference (30.1%) (Fig. 6D). In the 102°–106°E zone, geographic distance alone accounted for most of the explained variation in the turnover (40.9%) (Fig. 6E).

The effect of the differences in environmental heterogeneity on species turnover was limited. For the entire area of the Hengduan Mountains, difference in elevation range accounted for an extra 0.2% of the variation in the similarity when it was included in the regression model. Its additional effects on the species turnover in both the east–west (21°–26°N zone: 0.7%; 26°–30°N zone: 0.4%; and 30°–35°N zone: 0.3%) and south–north (98°–102°E zone: 0.1%; and 102°–106°E zone: 0.3%) directions were also small. Only a small amount of the variation in the similarity was additionally explained by difference in county area (entire Hengduan Mountains: 0.6%; 21°–26°N zone: 0.2%; 26°–30°N zone: 3.5%; 30°–35°N zone: 0.1%; 98°–102°E zone: 0.2%; and 102°–106°E zone: 0.1%), showing that county size did not strongly influence the patterns. Full details of the multiple regressions are provided in Appendix S7.

## Discussion

### Species turnover patterns of nonvolant small mammals in the Hengduan Mountains

The present study specifically explores the species turnover of nonvolant small mammals in a typical and uniquely characteristic mountainous region. With an elevation range of approximately 7500 m and a wealth of habitat variation, including dry‐hot valleys, glaciers, natural forests, and steppes (Wu et al. 2013), few comparable sites are found even globally. By examining spatial patterns in both longitudinal and latitudinal directions and along elevational gradients, our study indicates that the expected distance decay relationship for small mammals is evident in the Hengduan Mountains. At the global and continental scales, it has been widely recognized that the species turnover of vertebrates is particularly high in mountainous regions (Buckley and Jetz 2008; Melo et al. 2009; Dobrovolski et al. 2012). This is because mountainous regions typically harbor a wide gradient of habitats over short distances and because high mountains often serve as insurmountable dispersal barriers. In keeping with previous studies, mammalian turnover is demonstrated to be rapid in the Hengduan Mountains, and there is a nearly complete species replacement between the farthest pair of counties (spanning 1474 km, Jaccard similarity = 0.015), which are located at the southeast and northwest ends of this region, respectively. The mammalian fauna of the southeast Hengduan Mountains consists of a variety of species that have adapted to the subtropical humid forests, such as the striped squirrels and white‐bellied rats, whereas the fauna in the northwest regions is dominated by temperate steppe mammals such as pikas, marmots, and voles. Usually, such a high rate of turnover is typical at the continental scale or for individual montane gradients (Presley et al. 2012). In central Sichuan, in spite of their proximity, two adjacent counties showed very dissimilar mammalian communities, which might be related to their locations on two opposing mountain slopes (i.e., the east and west slopes of Gongga Mountain, which differ greatly in climatic and vegetation conditions). Our study therefore confirms that mountainous regions may exhibit more complicated spatial patterns of species turnover than other landforms (e.g., flatlands).

### Dispersal and niche processes

One of our major aims was to distinguish the effects of dispersal limitation and niche limitation in generating the species turnover patterns of nonvolant small mammals in the Hengduan Mountains. Our results specifically support the widely held view that both processes are important determinants, but in the Hengduan Mountains, dispersal processes play a primary role. The findings are inconsistent with the results of Qian et al. (2009), where the turnover patterns of mammalian turnover in North America are mainly driven by the joint effect of two processes. Such a discrepancy may be explained by two reasons. One reason is that the study objects of Qian et al. (2009) included bats and large mammals for which dispersal is less limiting, and the other reason is that the Hengduan Mountains comprises a smaller area with shorter environmental gradients than does North America.

Dispersal limitation can affect the community similarity by three major mechanisms: limited dispersal abilities of species, geographic barriers to dispersal, and environmental barriers to dispersal. For species turnover generated by limited dispersal abilities, the rationale is that less vagile species are unable to colonize all environmentally appropriate areas (Heino et al. 2015). With the decrease in dispersal ability, organisms tend to have more restricted distributions and thus show higher turnover in species composition (Nekola and White 1999; Soininen et al. 2007). For example, insectivores (Erinaceomorpha and Soricomorpha) are poorer dispersers than Glires (Rodentia and Lagomorpha) due to their generally smaller body sizes, inferior eyesight and hearing ability (Gliwicz and Taylor 2002; Schloss et al. 2012), and different food habits (insectivores, as typical carnivorous mammals normally have lower dispersal abilities than herbivores and omnivores do, Munguía et al. 2008). It is more difficult for insectivores to disperse into the same distant, suitable habitats in the Hengduan Mountains within a given time period. Our results also showed that the species similarity of insectivores decreased with geographic distance at a higher rate than Glires (Appendix S5, Table S2 and Figure S2), providing some evidence that limited dispersal abilities may play a pivotal role in shaping the turnover patterns here.

Other than the dispersal ability per se, the unique spatial configuration of the Hengduan Mountains is likely to explain much of the geographic variation in species composition by controlling the dispersal rate of species among sites. The effect is especially apparent in the directional turnover patterns, and species turnover is generally faster in the east–west direction than in the south–north direction for the regions at higher latitudes. This corresponds to the foremost geographic feature of the Hengduan Mountains – major mountain ranges and rivers running south to north, which mainly obstruct the dispersal of species between the east and west (Zhang et al. 1997b). However, it is worth noting that the species turnover in the 21°–26°N zone is instead slower than the south–north turnover, presumably due to the relatively small topographic relief along the longitudinal gradient in this zone (Fig. 1B). Our results are in line with the findings for Eastern Asian angiosperms (Qian et al. 2005) and Chinese woody plants (Wang et al. 2012), which suggest that the landscape configuration can strongly influence spatial turnover patterns.

Not only the topography but also the environments can provide barriers to the dispersal of organisms and cause species turnover (Wang et al. 2013). In the Hengduan Mountains, suitable habitat patches on a mountain may be separated by nonsuitable environments, with environmental conditions impeding dispersal (e.g., some lowland small mammal species are unable to reach the suitable habitats on the other side of a mountain because they cannot tolerate the harsh alpine environments, not because they cannot move that far). In this case, the compositional difference between sites is primarily due to environmental barriers and is not based on the dispersal ability or geographic barriers as a process. For some specific groups of small mammals, the connectivity of suitable habitats could be the most important determinant of the community similarity. For example, the dispersal of squirrels depends heavily on trees (Lurz et al. 1997), so it is difficult for them to reach all of the suitable sites in the absence of forest corridors.

Although environmental distance independently accounted for a small fraction of the variation in the similarity, it could not be simply concluded that niche processes have a negligible contribution to the patterns. However, the effects of niche processes on species turnover are mainly interrelated with those of other factors, such as the spatial processes (Fig. 5), because many environmental factors (temperature, precipitation, and PET) are strongly spatially structured in the Hengduan Mountains. Moreover, the fraction explained purely by geographic distance is associated with the dispersal ability or landscape configuration, but spatially structured environmental factors, which were not examined in this study (e.g., plant richness and vegetation coverage), may also contribute to this partition (Peres‐Neto et al. 2012).

### Geographic isolation and historical processes

Our results clearly indicated that geographic isolation is relevant to the current patterns of species turnover of small mammal in the Hengduan Mountains. The collision between India and Asia approximately 40 to 50 million years ago gave rise to many major geologic events, including the uplift of the Qinghai–Tibetan Plateau and the Hengduan Mountains. This movement has yielded a large number of high mountains (>5000 m) and deeply carved valleys, thereby creating a high number of isolated regions for allopatric speciation (Shi et al. 1998). Multiple lines of evidence have demonstrated that a substantial amount of the extant nonvolant small mammals in this region owe their origins to this orogeny, such as the genus *Eothenomys* (Luo et al. 2004) and *Apodemus* (Fan et al. 2011). After a long period, many species are endemic to a few mountain ranges, which ultimately leads to high regional turnover. Additionally, once allopatric speciation events have occurred, many subsequent effects may help maintain the spatial variation in species composition. For example, priority effects may manifest, in which the species that already occupy a niche repel an invading species and prevent the establishment of populations (Chase 2007). The importance of geographic isolation in the Hengduan Mountains is further confirmed by the increasing turnover toward the higher‐elevation zones. As altitude rises, the increased degree of isolation may result in the reduced dispersal and the lower rates of colonization in mountain ranges (i.e., ‘montane islands,’ Brown 1971), which in turn cause the higher differentiation in mammal assemblages.

Historical processes are among the most acknowledged but least understood mechanisms of species turnover (Baselga 2010; Leprieur et al. 2011). The Pleistocene glacial history of the Hengduan Mountains is possibly an important driver of the turnover patterns, and this history can act via two distinct mechanisms. During the ice age, alpine glaciers were widespread throughout the high‐elevation areas of the Hengduan Mountains, whereas most lowland regions, such as deep valleys and rift basins, were unglaciated and the tropical and subtropical habitats were retained (Zhang 2002; Chen et al. 2010). As a result, the species turnover between the low and high‐elevation areas can be directly produced by the selective extinctions of montane species with small ranges due to the harshness of the environment (Davies et al. 2009; Leprieur et al. 2011). For the second potential mechanism, the numerous isolated lowland regions provided a network of glacial refugia for the vagile species retreating from higher elevations and were the centers of subsequent speciation. After the retreat of glaciers, these areas became the pumps from which the surviving and newly evolved species were able to recolonize the high‐elevation areas (Lei et al. 2015). The glacial–interglacial cycles occurred many times in the Hengduan Mountains during the Pleistocene, which may be largely responsible for the rapid spatial turnover through repeated shifts in the distributions of small mammals and the unceasing emergence of evolutionary novelties (Chen et al. 2010; Fan et al. 2011). Importantly, the historical climate may have contributed greatly to current species turnover patterns in the Hengduan Mountains.

### Conclusions and implications for biodiversity conservation

In summary, our results highlight the integrated effects of dispersal, niche, and isolation processes on the species turnover patterns of nonvolant small mammals in the Hengduan Mountains of China. The directional turnover patterns generally fit the geographic structure of the region and suggest that the spatial configuration of the landscape can strongly influence the spatial turnover in mountainous regions. The increasing trend of spatial turnover toward the higher‐elevation zones clearly indicates the important role of geographic isolation in determining the species turnover rate in mountainous regions.

The spatial turnover patterns found in this study provide valuable information for designing regional reserve networks. In the areas with high turnover, the total biodiversity should be captured by a single sufficiently sized reserve or by a set of smaller reserves (Wiersma and Urban 2005). The extremely high species turnover in the Hengduan Mountains, both in the east–west and south–north directions, ideally calls for many reserves that are associated with each area of endemism to optimize the conservation of small mammal diversity in this region. That is, a connected reserve network consisting of a sufficient number of small‐ and moderate‐sized reserves should be established to protect the distinct mammalian communities in different areas, with the endemic species in local communities and their habitats conserved (Rickbeil et al. 2014). In practice, however, systematic conservation planning must also account for factors such as connectivity, costs, and explicit target species. Consequently, we suggest that central Sichuan and southwestern Yunnan, which have the highest levels of species turnover and the most unique species representation, are the priority conservation areas of the Hengduan Mountains.

## Conflict of Interest

None declared.

## Supporting information


**Appendix S1**. Species list of non‐volant small mammals in the Hengduan Mountains.

**Appendix S2.** Presence‐absence dataset of small mammals in 164 counties of the Hengduan Mountains (available from the Dryad Digital Repository: http://dx.doi.org/10.5061/dryad.7qb11).

**Appendix S3.** Habitat types in the Hengduan Mountains (Table S1) and the results of principal component analysis of environmental variables (Table S2).

**Appendix S4**. R codes of the simple and partial Mantel tests and multiple regressions on distance matrices (MRM) conducted using the R package ‘ecodist’.

**Appendix S5.** Comparison of the halving distances between Glires and insectivores in the entire area of the Hengduan Mountains (Table S1), the halving distance of each mammal group was calculated for the linear and logarithmic regression models between Jaccard similarity and geographic distance (km) (Figure S1); comparison of the halving distances of all non‐volant small mammal species among four elevation zones of the Hengduan Mountains (Table S2), the halving distance in each elevation zone was calculated for the linear, logarithmic and exponential regression models between Simpson similarity and geographic distance (km) (Figure S2).

**Appendix S6.** Results of the partial Mantel tests examining the correlations between the Jaccard similarity of non‐volant small mammals and the four explanatory factors in the entire area of the Hengduan Mountains (Table S1) and in the five longitudinal/latitudinal (21°–26°N: Table S2; 26°–30°N: Table S3; 30°–35°N: Table S4; 98°–102°E: Table S5; 102°–106°E: Table S6) zones of the region.

**Appendix S7.** Coefficients of determination (*R* ^2^) for the multiple regression analyses showing the contributions of the four explanatory matrices and the difference in area matrix to explaining the variation in the Jaccard similarity matrix of non‐volant small mammals in the entire area of the Hengduan Mountains and in the five longitudinal/latitudinal zones of the region.
