# Supplementary material for: Dispersal, niche, and isolation processes jointly explain species turnover patterns of nonvolant small mammals in a large mountainous region of China
Source: Ecol Evol. 2016 Jan 18;6(4):946–60. doi: 10.1002/ece3.1962 (PMC4761768; doi:10.1002/ece3.1962)
Supplement: Supplementary file 1 — Appendix S1. Species list of non‐volant small mammals in the Hengduan Mountains. [file ECE3-6-0946-s001.doc]

***Ecology and Evolution***

**Dispersal, niche and isolation processes** **jointly explain species turnover patterns of non-volant small mammals in a large mountainous region of China**

Zhixin Wen, Qing Quan, Yuanbao Du, Lin Xia, Deyan Ge and Qisen Yang*

*Corresponding author: Key Laboratory of Zoological Systematics and Evolution, Institute of Zoology, Chinese Academy of Sciences, 1 Beichen West Road, Beijing, 100101, China;

yangqs@ioz.ac.cn; telephone: +86-010-64807225

**Appendix S1**

**Species list of non-volant small mammals in the Hengduan Mountains**

**Table S1.** The species list of non-volant small mammals in the Hengduan Mountains based on the taxonomic system of *Mammal Species of the World*. There are totally 191 species, belonging to 5 orders, 14 families and 80 genera.

| **Orders** | **Families** | | **Genera** | | **Scientific Names** | |
| --- | --- | --- | --- | --- | --- | --- |
| Erinaceomorpha | Erinaceidae | | *Neotetracus* | | *Neotetracus sinensis* | |
| Erinaceomorpha | Erinaceidae | | *Erinaceus* | | *Erinaceus amurensis* | |
| Erinaceomorpha | Erinaceidae | | *Mesechinus* | | *Mesechinus hughi* | |
| Erinaceomorpha | Erinaceidae | | *Hemiechinus* | | *Hemiechinus auritus* | |
| Erinaceomorpha | Erinaceidae | | *Hylomys* | | *Hylomys suillus* | |
| Soricomorpha | Soricidae | | *Sorex* | | *Sorex minutus* | |
| Soricomorpha | Soricidae | | *Sorex* | | *Sorex caecutiens* | |
| Soricomorpha | Soricidae | | *Sorex* | | *Sorex araneus* | |
| Soricomorpha | Soricidae | | *Sorex* | | *Sorex cylindricauda* | |
| Soricomorpha | Soricidae | | *Sorex* | | *Sorex bedfordiae* | |
| Soricomorpha | Soricidae | | *Sorex* | | *Sorex sinalis* | |
| Soricomorpha | Soricidae | | *Sorex* | | *Sorex thibetanus* | |
| Soricomorpha | Soricidae | | *Sorex* | | *Sorex excelsus* | |
| Soricomorpha | Soricidae | | *Sorex* | | *Sorex cansulus* | |
| Soricomorpha | Soricidae | | *Sorex* | | *Sorex minutissimus* | |
| **Orders** | **Families** | | **Genera** | | **Scientific Names** | |
| Soricomorpha | Soricidae | | *Soriculus* | | *Soriculus nigrescens* | |
| Soricomorpha | Soricidae | | *Episoriculus* | | *Episoriculus caudatus* | |
| Soricomorpha | Soricidae | | *Episoriculus* | | *Episoriculus leucops* | |
| Soricomorpha | Soricidae | | *Episoriculus* | | *Episoriculus macrurus* | |
| Soricomorpha | Soricidae | | *Blarinella* | | *Blarinella quadraticauda* | |
| Soricomorpha | Soricidae | | *Suncus* | | | *Suncus murinus* |
| Soricomorpha | Soricidae | | *Suncus* | | | *Suncus stoliczkanus* |
| Soricomorpha | Soricidae | | *Suncus* | | | *Suncus etruscus* |
| Soricomorpha | Soricidae | | *Chodsigoa* | | | *Chodsigoa salenskii* |
| Soricomorpha | Soricidae | | *Chodsigoa* | | | *Chodsigoa parva* |
| Soricomorpha | Soricidae | | *Chodsigoa* | | | *Chodsigoa lamula* |
| Soricomorpha | Soricidae | | *Chodsigoa* | | | *Chodsigoa hypsibia* |
| Soricomorpha | Soricidae | | *Chodsigoa* | | | *Chodsigoa parca* |
| Soricomorpha | Soricidae | | *Chodsigoa* | | | *Chodsigoa smithii* |
| Soricomorpha | Soricidae | | *Chodsigoa* | | | *Chodsigoa caovansunga* |
| Soricomorpha | Soricidae | | *Crocidura* | | | *Crocidura suaveolens* |
| Soricomorpha | Soricidae | | *Crocidura* | | | *Crocidura horsfieldii* |
| Soricomorpha | Soricidae | | *Crocidura* | | | *Crocidura russula* |
| Soricomorpha | Soricidae | | *Crocidura* | | | *Crocidura shantungensis* |
| Soricomorpha | Soricidae | | *Crocidura* | | | *Crocidura attenuata* |
| Soricomorpha | Soricidae | | *Crocidura* | | | *Crocidura dracula* |
| Soricomorpha | Soricidae | | *Crocidura* | | | *Crocidura lasiura* |
| Soricomorpha | Soricidae | | *Crocidura* | | | *Crocidura fuliginosa* |
| Soricomorpha | Soricidae | | *Chimarrogale* | | | *Chimarrogale himalayica* |
| Soricomorpha | Soricidae | | *Chimarrogale* | | | *Chimarrogale styani* |
| Soricomorpha | Soricidae | | *Nectogale* | | | *Nectogale elegans* |
| Soricomorpha | Soricidae | | *Anourosorex* | | | *Anourosorex squamipes* |
| Soricomorpha | Talpidae | | *Uropsilus* | | | *Uropsilus soricipes* |
| **Orders** | **Families** | | **Genera** | | | **Scientific Names** |
| Soricomorpha | Talpidae | | *Uropsilus* | | | *Uropsilus gracilis* |
| Soricomorpha | Talpidae | | *Uropsilus* | | | *Uropsilus andersoni* |
| Soricomorpha | Talpidae | | *Scaptonyx* | | | *Scaptonyx fusicaudus* |
| Soricomorpha | Talpidae | | *Scaptochirus* | | | *Scaptochirus moschatus* |
| Soricomorpha | Talpidae | | *Euroscaptor* | | | *Euroscaptor grandis* |
| Soricomorpha | Talpidae | | *Euroscaptor* | | | *Euroscaptor micrura* |
| Soricomorpha | Talpidae | | *Euroscaptor* | | | *Euroscaptor longirostris* |
| Soricomorpha | Talpidae | | *Euroscaptor* | | | *Euroscaptor klossi* |
| Soricomorpha | Talpidae | | *Scapanulus* | | | *Scapanulus oweni* |
| Soricomorpha | Talpidae | | *Parascaptor* | | | *Parascaptor leucura* |
| Soricomorpha | Talpidae | | *Mogera* | | | *Mogera insularis* |
| Scandentia | Tupaiidae | | *Tupaia* | | | *Tupaia belangeri* |
| Lagomorpha | Leporidae | | *Lepus* | | | *Lepus oiostolus* |
| Lagomorpha | Leporidae | | *Lepus* | | | *Lepus tolai* |
| Lagomorpha | Leporidae | | *Lepus* | | | *Lepus comus* |
| Lagomorpha | Ochotonidae | | *Ochotona* | | | *Ochotona cansus* |
| Lagomorpha | Ochotonidae | | *Ochotona* | | | *Ochotona thibetana* |
| Lagomorpha | Ochotonidae | | *Ochotona* | | | *Ochotona forresti* |
| Lagomorpha | Ochotonidae | | *Ochotona* | | | *Ochotona curzoniae* |
| Lagomorpha | Ochotonidae | | *Ochotona* | | | *Ochotona roylei* |
| Lagomorpha | Ochotonidae | | *Ochotona* | | | *Ochotona macrotis* |
| Lagomorpha | Ochotonidae | | *Ochotona* | | | *Ochotona rutila* |
| Lagomorpha | Ochotonidae | | *Ochotona* | | | *Ochotona gloveri* |
| Lagomorpha | Ochotonidae | | *Ochotona* | | | *Ochotona dauurica* |
| Lagomorpha | Ochotonidae | | *Ochotona* | | | *Ochotona erythrotis* |
| Lagomorpha | Ochotonidae | | *Ochotona* | | | *Ochotona koslowi* |
| Lagomorpha | | Ochotonidae | | *Ochotona* | *Ochotona thomasi* | |
| Lagomorpha | | Ochotonidae | | *Ochotona* | *Ochotona muliensis* | |
| **Orders** | | **Families** | | **Genera** | **Scientific Names** | |
| Lagomorpha | | Ochotonidae | | *Ochotona* | *Ochotona huangensis* | |
| Rodentia | | Sciuridae | | *Belomys* | *Belomys pearsonii* | |
| Rodentia | | Sciuridae | | *Pteromys* | *Pteromys volans* | |
| Rodentia | | Sciuridae | | *Aeretes* | *Aeretes melanopterus* | |
| Rodentia | | Sciuridae | | *Petaurista* | *Petaurista petaurista* | |
| Rodentia | | Sciuridae | | *Petaurista* | *Petaurista elegans* | |
| Rodentia | | Sciuridae | | *Petaurista* | *Petaurista alborufus* | |
| Rodentia | | Sciuridae | | *Petaurista* | *Petaurista xanthotis* | |
| Rodentia | | Sciuridae | | *Petaurista* | *Petaurista yunnanensis* | |
| Rodentia | | Sciuridae | | *Petaurista* | *Petaurista philippensis* | |
| Rodentia | | Sciuridae | | *Trogopterus* | *Trogopterus xanthipes* | |
| Rodentia | | Sciuridae | | *Hylopetes* | *Hylopetes alboniger* | |
| Rodentia | | Sciuridae | | *Eupetaurus* | *Eupetaurus cinereus* | |
| Rodentia | | Sciuridae | | *Callosciurus* | *Callosciurus erythraeus* | |
| Rodentia | | Sciuridae | | *Callosciurus* | *Callosciurus quinquestriatus* | |
| Rodentia | | Sciuridae | | *Callosciurus* | *Callosciurus pygerythrus* | |
| Rodentia | | Sciuridae | | *Callosciurus* | *Callosciurus phayrei* | |
| Rodentia | | Sciuridae | | *Callosciurus* | *Callosciurus inornatus* | |
| Rodentia | | Sciuridae | | *Callosciurus* | *Callosciurus finlaysonii* | |
| Rodentia | | Sciuridae | | *Menetes* | *Menetes berdmorei* | |
| Rodentia | | Sciuridae | | *Tamiops* | *Tamiops maritimus* | |
| Rodentia | | Sciuridae | | *Tamiops* | *Tamiops macclellandi* | |
| Rodentia | | Sciuridae | | *Tamiops* | *Tamiops swinhoei* | |
| Rodentia | | Sciuridae | | *Dremomys* | *Dremomys pernyi* | |
| Rodentia | | Sciuridae | | *Dremomys* | *Dremomys lokriah* | |
| Rodentia | | Sciuridae | | *Dremomys* | *Dremomys rufigenis* | |
| Rodentia | | Sciuridae | | *Dremomys* | *Dremomys gularis* | |
| Rodentia | | Sciuridae | | *Dremomys* | *Dremomys pyrrhomerus* | |
| **Orders** | | **Families** | | **Genera** | **Scientific Names** | |
| Rodentia | | Sciuridae | | *Ratufa* | *Ratufa bicolor* | |
| Rodentia | | Sciuridae | | *Sciurotamias* | *Sciurotamias davidianus* | |
| Rodentia | | Sciuridae | | *Sciurotamias* | *Sciurotamias forresti* | |
| Rodentia | | Sciuridae | | *Eutamias* | *Eutamias sibiricus* | |
| Rodentia | | Sciuridae | | *Marmota* | *Marmota himalayana* | |
| Rodentia | | Sciuridae | | *Marmota* | *Marmota baibacina* | |
| Rodentia | | Gliridae | | *Chaetocauda* | *Chaetocauda sichuanensis* | |
| Rodentia | | Hystricidae | | *Hystrix* | *Hystrix brachyura* | |
| Rodentia | | Hystricidae | | *Atherurus* | *Atherurus macrourus* | |
| Rodentia | | Dipodidae | | *Sicista* | *Sicista concolor* | |
| Rodentia | | Dipodidae | | *Eozapus* | *Eozapus setchuanus* | |
| Rodentia | | Dipodidae | | *Dipus* | *Dipus sagitta* | |
| Rodentia | | Dipodidae | | *Cardiocranius* | *Cardiocranius paradoxus* | |
| Rodentia | | Dipodidae | | *Allactaga* | *Allactaga sibirica* | |
| Rodentia | | Platacanthomyidae | | *Typhlomys* | *Typhlomys cinereus* | |
| Rodentia | | Spalacidae | | *Rhizomys* | *Rhizomys pruinosus* | |
| Rodentia | | Spalacidae | | *Rhizomys* | *Rhizomys sinensis* | |
| Rodentia | | Spalacidae | | *Rhizomys* | *Rhizomys sumatrensis* | |
| Rodentia | | Spalacidae | | *Cannomys* | *Cannomys badius* | |
| Rodentia | | Spalacidae | | *Myospalax* | *Myospalax aspalax* | |
| Rodentia | | Spalacidae | | *Eospalax* | *Eospalax fontanierii* | |
| Rodentia | | Spalacidae | | *Eospalax* | *Eospalax rufescens* | |
| Rodentia | | Spalacidae | | *Eospalax* | *Eospalax rothschildi* | |
| Rodentia | | Spalacidae | | *Eospalax* | *Eospalax smithii* | |
| Rodentia | | Muridae | | *Vernaya* | *Vernaya fulva* | |
| Rodentia | | Muridae | | *Vandeleuria* | *Vandeleuria oleracea* | |
| Rodentia | | Muridae | | *Chiropodomys* | *Chiropodomys gliroides* | |
| Rodentia | | Muridae | | *Chiromyscus* | *Chiromyscus chiropus* | |
| **Orders** | | **Families** | | **Genera** | **Scientific Names** | |
| Rodentia | | Muridae | | *Bandicota* | *Bandicota indica* | |
| Rodentia | | Muridae | | *Apodemus* | *Apodemus draco* | |
| Rodentia | | Muridae | | *Apodemus* | *Apodemus latronum* | |
| Rodentia | | Muridae | | *Apodemus* | *Apodemus peninsulae* | |
| Rodentia | | Muridae | | *Apodemus* | *Apodemus agrarius* | |
| Rodentia | | Muridae | | *Apodemus* | *Apodemus chevrieri* | |
| Rodentia | | Muridae | | *Dacnomys* | *Dacnomys millardi* | |
| Rodentia | | Muridae | | *Hadromys* | *Hadromys yunnanensis* | |
| Rodentia | | Muridae | | *Hapalomys* | *Hapalomys longicaudatus* | |
| Rodentia | | Muridae | | *Rattus* | *Rattus rattus* | |
| Rodentia | | Muridae | | *Rattus* | *Rattus tanezumi* | |
| Rodentia | | Muridae | | *Rattus* | *Rattus nitidus* | |
| Rodentia | | Muridae | | *Rattus* | *Rattus pyctoris* | |
| Rodentia | | Muridae | | *Rattus* | *Rattus losea* | |
| Rodentia | | Muridae | | *Rattus* | *Rattus norvegicus* | |
| Rodentia | | Muridae | | *Leopoldamys* | *Leopoldamys edwardsi* | |
| Rodentia | | Muridae | | *Maxomys* | *Maxomys surifer* | |
| Rodentia | | Muridae | | *Maxomys* | *Maxomys rajah* | |
| Rodentia | | Muridae | | *Mus* | *Mus musculus* | |
| Rodentia | | Muridae | | *Mus* | *Mus pahari* | |
| Rodentia | | Muridae | | *Mus* | *Mus caroli* | |
| Rodentia | | Muridae | | *Mus* | *Mus cookii* | |
| Rodentia | | Muridae | | *Mus* | *Mus cervicolor* | |
| Rodentia | | Muridae | | *Mus* | *Mus vulcani* | |
| Rodentia | | Muridae | | *Berylmys* | *Berylmys bowersi* | |
| Rodentia | | Muridae | | *Berylmys* | *Berylmys berdmorei* | |
| Rodentia | | Muridae | | *Berylmys* | *Berylmys manipulus* | |
| Rodentia | | Muridae | | *Niviventer* | *Niviventer eha* | |
| **Orders** | | **Families** | | **Genera** | **Scientific Names** | |
| Rodentia | | Muridae | | *Niviventer* | *Niviventer cremoriventer* | |
| Rodentia | | Muridae | | *Niviventer* | *Niviventer fulvescens* | |
| Rodentia | | Muridae | | *Niviventer* | *Niviventer andersoni* | |
| Rodentia | | Muridae | | *Niviventer* | *Niviventer niviventer* | |
| Rodentia | | Muridae | | *Niviventer* | *Niviventer excelsior* | |
| Rodentia | | Muridae | | *Niviventer* | *Niviventer brahma* | |
| Rodentia | | Muridae | | *Micromys* | *Micromys minutus* | |
| Rodentia | | Cricetidae | | *Cricetulus* | *Cricetulus longicaudatus* | |
| Rodentia | | Cricetidae | | *Cricetulus* | *Cricetulus kamensis* | |
| Rodentia | | Cricetidae | | *Cricetulus* | *Cricetulus migratorius* | |
| Rodentia | | Cricetidae | | *Cansumys* | *Cansumys canus* | |
| Rodentia | | Cricetidae | | *Alticola* | *Alticola stoliczkanus* | |
| Rodentia | | Cricetidae | | *Microtus* | *Microtus arvalis* | |
| Rodentia | | Cricetidae | | *Microtus* | *Microtus fortis* | |
| Rodentia | | Cricetidae | | *Microtus* | *Microtus oeconomus* | |
| Rodentia | | Cricetidae | | *Microtus* | *Microtus clarkei* | |
| Rodentia | | Cricetidae | | *Microtus* | *Microtus agrestis* | |
| Rodentia | | Cricetidae | | *Microtus* | *Microtus limnophilus* | |
| Rodentia | | Cricetidae | | *Lasiopodomys* | *Lasiopodomys fuscus* | |
| Rodentia | | Cricetidae | | *Volemys* | *Volemys musseri* | |
| Rodentia | | Cricetidae | | *Volemys* | *Volemys millicens* | |
| Rodentia | | Cricetidae | | *Proedromys* | *Proedromys bedfordi* | |
| Rodentia | | Cricetidae | | *Proedromys* | *Proedromys liangshanensis* | |
| Rodentia | | Cricetidae | | *Phaiomys* | *Phaiomys leucurus* | |
| Rodentia | | Cricetidae | | *Neodon* | *Neodon irene* | |
| Rodentia | | Cricetidae | | *Neodon* | *Neodon sikimensis* | |
| Rodentia | | Cricetidae | | *Caryomys* | *Caryomys eva* | |
| Rodentia | | Cricetidae | | *Caryomys* | *Caryomys inez* | |
| **Orders** | | **Families** | | **Genera** | **Scientific Names** | |
| Rodentia | | Cricetidae | | *Eothenomys* | *Eothenomys melanogaster* | |
| Rodentia | | Cricetidae | | *Eothenomys* | *Eothenomys miletus* | |
| Rodentia | | Cricetidae | | *Eothenomys* | *Eothenomys olitor* | |
| Rodentia | | Cricetidae | | *Eothenomys* | *Eothenomys cachinus* | |
| Rodentia | | Cricetidae | | *Eothenomys* | *Eothenomys custos* | |
| Rodentia | | Cricetidae | | *Eothenomys* | *Eothenomys proditor* | |
| Rodentia | | Cricetidae | | *Eothenomys* | *Eothenomys chinensis* | |
| Rodentia | | Cricetidae | | *Eothenomys* | *Eothenomys wardi* | |
