# Supplementary material for: Dispersal, niche, and isolation processes jointly explain species turnover patterns of nonvolant small mammals in a large mountainous region of China
Source: Ecol Evol. 2016 Jan 18;6(4):946–60. doi: 10.1002/ece3.1962 (PMC4761768; doi:10.1002/ece3.1962)
Supplement: Supplementary file 3 — Appendix S3. Habitat types in the Hengduan Mountains (Table S1) and the results of principal component analysis of environmental variables (Table S2). [file ECE3-6-0946-s003.doc]

***Ecology and Evolution***

**Dispersal, niche and isolation processes** **jointly explain species turnover patterns of non-volant small mammals in a large mountainous region of China**

Zhixin Wen, Qing Quan, Yuanbao Du, Lin Xia, Deyan Ge and Qisen Yang*

*Corresponding author: Key Laboratory of Zoological Systematics and Evolution, Institute of Zoology, Chinese Academy of Sciences, 1 Beichen West Road, Beijing, 100101, China;

yangqs@ioz.ac.cn; telephone: +86-010-64807225

**Appendix S3**

**Habitat types in the Hengduan Mountains (Table S1) and the results of principal component analysis of environmental variables (Table S2)**

**Table S1.** Main habitat types in the Hengduan Mountains.

| **Habitat type** |
| --- |
| (1) Tropical rainforest |
| (2) Dry-hot valley shrubland  (3) Steppe |
| (4) Evergreen broadleaf forest |
| (5) Evergreen and deciduous mixed broadleaf forest |
| (6) Coniferous and deciduous broadleaf mixed forest |
| (7) Coniferous forest |
| (8) Subalpine shrub and meadow  (9) Alpine meadow |

**Table S2.** Results of the principal component analysis (PCA) of the nine environmental variables in the Hengduan Mountains.

|  | **PC1** | **PC2** | **PC3** | **PC4** |
| --- | --- | --- | --- | --- |
| **PCA results** |  |  |  |  |
| Eigenvalue | 6.29 | 1 | 0.72 | 0.53 |
| Percent of variance (%) | 69.8 | 11.1 | 8 | 5.9 |
| Cumulative Percent of variance (%) | 69.8 | 81 | 89 | 94.9 |
| **Correlation coefficient** |  |  |  |  |
| MAT | 0.975 | -0.039 | 0.002 | 0.209 |
| MWT | 0.924 | -0.002 | -0.043 | 0.347 |
| MCT | 0.983 | -0.055 | 0.034 | 0.15 |
| TS | -0.912 | 0.122 | -0.139 | 0.153 |
| MAP | 0.795 | 0.368 | 0.2 | -0.029 |
| PS | -0.424 | -0.74 | 0.493 | 0.105 |
| PET | 0.973 | -0.137 | 0.039 | 0.057 |
| NDVI | 0.787 | 0.029 | 0.269 | -0.514 |
| NHT | -0.552 | 0.527 | 0.584 | 0.208 |

Variable abbreviations: MAT, mean annual temperature (°C); MWT, mean temperature of the warmest month (°C); MCT, mean temperature of the coldest month (°C); TS, temperature seasonality (°C); MAP, mean annual precipitation (mm) ; PS, precipitation seasonality (mm); PET, mean annual potential evapotranspiration (mm); NDVI, normalized difference vegetation index ; NHT, number of habitat types.

Data resource: MAT, MWT, MCT, MAP and PS data were from the WorldClim Dataset (version 1.4, http://www.worldclim.org/) (Hijmans et al. 2005); PET data were from the CGIAR-CSI Global PET Database (http://www.cgiar-csi.org/) (Zorner et al. 2008); NDVI data were from http://www.data.ac.cn/.

**References**

Hijmans RJ, Cameron SE, Parra JL, Jones PG, Jarvis A (2005) Very high resolution interpolated climate surfaces for global land areas. International Journal of Climatology 25:1965-1978.

Zorner RJ, Trabucco A, Bossio DA, Verchot LV (2008) Climate change mitigation: A spatial analysis of global land suitability for clean development mechanism afforestation and reforestation. Agriculture, Ecosystems & Environment 126:67-80.
