# Supplementary material for: Dispersal, niche, and isolation processes jointly explain species turnover patterns of nonvolant small mammals in a large mountainous region of China
Source: Ecol Evol. 2016 Jan 18;6(4):946–60. doi: 10.1002/ece3.1962 (PMC4761768; doi:10.1002/ece3.1962)
Supplement: Supplementary file 4 — Appendix S4. R codes of the simple and partial Mantel tests and multiple regressions on distance matrices (MRM) conducted using the R package ‘ecodist’. [file ECE3-6-0946-s004.doc]

***Ecology and Evolution***

**Dispersal, niche and isolation processes** **jointly explain species turnover patterns of non-volant small mammals in a large mountainous region of China**

Zhixin Wen, Qing Quan, Yuanbao Du, Lin Xia, Deyan Ge and Qisen Yang*

*Corresponding author: Key Laboratory of Zoological Systematics and Evolution, Institute of Zoology, Chinese Academy of Sciences, 1 Beichen West Road, Beijing, 100101, China;

yangqs@ioz.ac.cn; telephone: +86-010-64807225

**Appendix S4**

**R codes of the simple and partial Mantel tests and multiple regressions on distance matrices (MRM) conducted using the R package ‘ecodist’**

**Simple and partial Mantel tests:**

Read data:

> library(ecodist)

> JSDIS<-as.dist(read.table("jaccard similarity.txt"))

> GDDIS<-as.dist(read.table("geographic distance.txt"))

> EDDIS<-as.dist(read.table("environmental distance.txt"))

> DAEDIS<-as.dist(read.table("difference in average elevation.txt"))

> DERDIS<-as.dist(read.table("difference in elevation range.txt"))

Simple mantel tests (simple mantel test of the relationship between JSDIS and each explanatory matrix, number of permutations = 1,000):

> mantel(JSDIS~GDDIS,nperm=1,000)

> mantel(JSDIS~EDDIS,nperm=1,000)

> mantel(JSDIS~DAEDIS,nperm=1,000)

> mantel(JSDIS~DERDIS,nperm=1,000)

Partial mantel tests (partial mantel test of the relationship between JSDIS and each explanatory matrix while controling the effect of a third matirx, number of permutations = 1,000):

>mantel(JSDIS~GDDIS+EDDIS,nperm=1,000)

>mantel(JSDIS~GDDIS+ DAEDIS,nperm=1,000)

>mantel(JSDIS~GDDIS+ DERDIS,nperm=1,000)

>mantel(JSDIS~EDDIS+GDDIS,nperm=1,000)

>mantel(JSDIS~EDDIS+ DAEDIS,nperm=1,000)

>mantel(JSDIS~EDDIS+ DERDIS,nperm=1,000)

>mantel(JSDIS~ DAEDIS+GDDIS,nperm=1,000)

>mantel(JSDIS~ DAEDIS+EDDIS,nperm=1,000)

>mantel(JSDIS~ DAEDIS+DERDIS,nperm=1,000)

>mantel(JSDIS~ DERDIS+GDDIS,nperm=1,000)

>mantel(JSDIS~ DERDIS+EDDIS,nperm=1,000)

>mantel(JSDIS~ DERDIS+DAEDIS,nperm=1,000)

**Multiple regressions on distance matrices (MRM)**

Read data:

> library(ecodist)

> JSDIS<-as.dist(read.table("jaccard similarity.txt"))

> GDDIS<-as.dist(read.table("geographic distance.txt"))

> EDDIS<-as.dist(read.table("environmental distance.txt"))

> DAEDIS<-as.dist(read.table("difference in average elevation.txt"))

> DERDIS<-as.dist(read.table("difference in elevation range.txt"))

> DADIS<-as.dist(read.table("difference in area.txt"))

Multiple regressions on distance matrices (MRM) using permutation tests (n=1,000) of significance for regression coefficients and *R*^2^:

> MRM(JSDIS~GDDIS,nperm=1,000)

> MRM(JSDIS~GDDIS,nperm=1,000)

> MRM(JSDIS~EDDIS,nperm=1,000)

> MRM(JSDIS~DAEDIS,nperm=1,000)

> MRM(JSDIS~ DERDIS,nperm=1,000)

> MRM(JSDIS~GDDIS+ EDDIS,nperm=1,000)

> MRM(JSDIS~GDDIS + DAEDIS,nperm=1,000)

> MRM(JSDIS~ EDDIS+ DAEDIS,nperm=1,000)

> MRM(JSDIS~GDDIS+ EDDIS+ DAEDIS,nperm=1,000)

> MRM(JSDIS~GDDIS+ EDDIS+ DAEDIS+DERDIS,nperm=1,000)

> MRM(JSDIS~GDDIS+ EDDIS+ DAEDIS+DERDIS+ DADIS,nperm=1,000)
