# Supplementary material for: Dispersal, niche, and isolation processes jointly explain species turnover patterns of nonvolant small mammals in a large mountainous region of China
Source: Ecol Evol. 2016 Jan 18;6(4):946–60. doi: 10.1002/ece3.1962 (PMC4761768; doi:10.1002/ece3.1962)

***Ecology and Evolution***

**Dispersal, niche and isolation processes** **jointly explain species turnover patterns of non-volant small mammals in a large mountainous region of China**

Zhixin Wen, Qing Quan, Yuanbao Du, Lin Xia, Deyan Ge and Qisen Yang*

*Corresponding author: Key Laboratory of Zoological Systematics and Evolution, Institute of Zoology, Chinese Academy of Sciences, 1 Beichen West Road, Beijing, 100101, China;

yangqs@ioz.ac.cn; telephone: +86-010-64807225

**Appendix S5**

**Comparison of the halving distances between Glires and insectivores in the entire area of the Hengduan Mountains (Table S1), the halving distance of each mammal group was calculated for the linear and logarithmic regression models between Jaccard similarity and geographic distance (km) (Figure S1); comparison of the halving distances of all non-volant small mammal species among four elevation zones of the Hengduan Mountains (Table S2), the halving distance in each elevation zone was calculated for the linear, logarithmic and exponential regression models between Simpson similarity and geographic distance (km) (Figure S2)**

**Table S1.** Halving distances (HD, the geographic distance that halves the similarity from its initial value) (km) of Glires (Rodentia and Lagomorpha) and insectivores (Erinaceomorpha and Soricomorpha) in the entire area of the Hengduan Mountains. The halving distance was calculated for the linear (y = ax +b) and logarithmic (y = alnx + b) regression models between Jaccard similarity of each mammal group and geographic distance (km), and each regression was performed with 1,000 permutations to determine the statistical significance (*P-*values, all < 0.001). The parameters (a, b) for each regression were given (note: the relationship between Jaccard similarity of insectivores and geographic distance could not be approximated by an exponential model).

| Mammal group | Initial  similarity | n | Linear | | | |  | Logarithmic | | | |
| --- | --- | --- | --- | --- | --- | --- | --- | --- | --- | --- | --- |
|  |  |  | a | b | *R^2^* | HD |  | a | b | *R^2^* | HD |
| Glires | 1 | 13,366 | -0.00063 | 0.7817 | 0.797 | 447 |  | -0.2450 | 1.9457 | 0.764 | 365 |
| Insectivores | 1 | 13,366 | -0.00062 | 0.6726 | 0.566 | 278 |  | -0.2549 | 1.8979 | 0.595 | 241 |

**Figure S1.** Relationship between the Jaccard similarity of (a) Glires and (b) insectivores and geographic distance (km) for all pairwise counties (*n* = 13,366) in the entire area of the Hengduan Mountains. The lines fitted indicate the linear and logarithmic regressions and all the regressions were significant at *P* < 0.001.


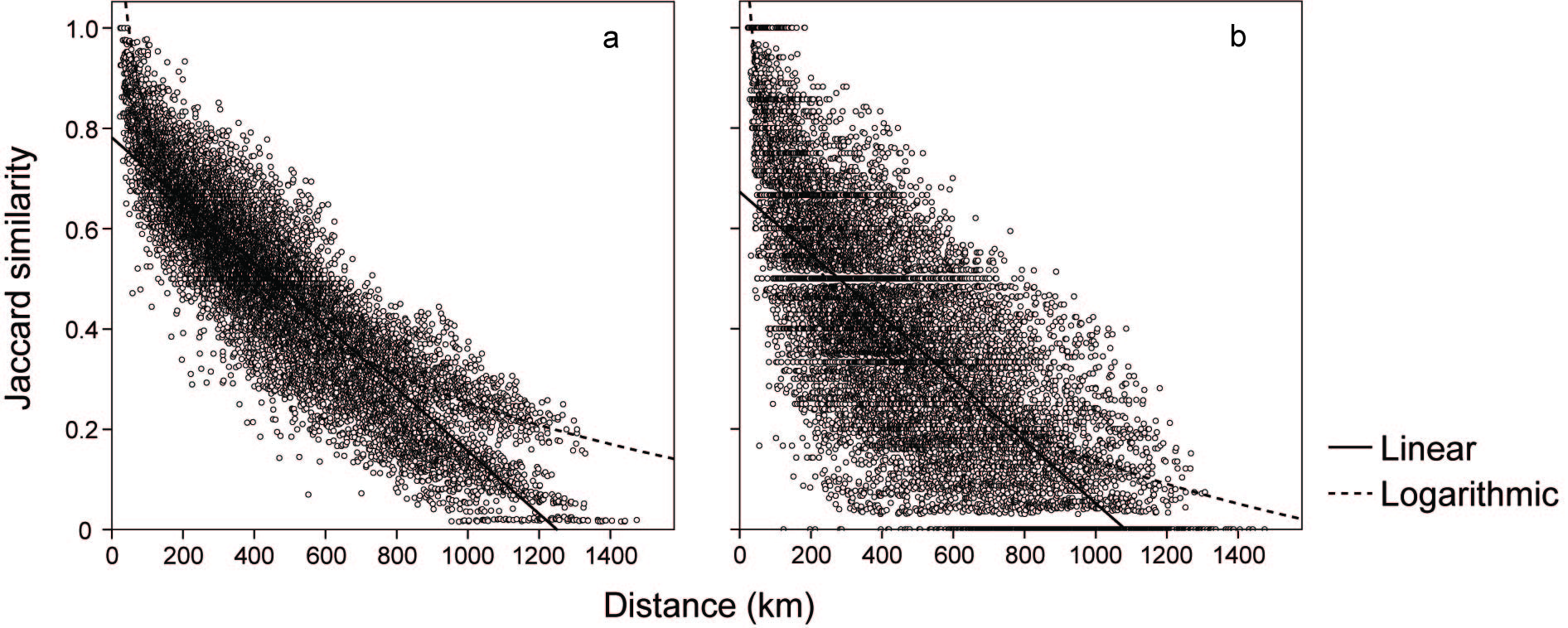


**Table S2.** Halving distances (HD, the geographic distance that halves the similarity from its initial value) (km) in the four elevation zones of the Hengduan Mountains. The halving distance was calculated for the linear (y = ax +b), logarithmic (y = alnx + b) and exponential (y = be^ax^) regression models between Simpson similarity of non-volant small mammals and geographic distance (km) in each elevation zone, and each regression was performed with 1,000 permutations to determine the statistical significance (*P-*values, all < 0.001). The parameters (a, b) for each regression were given.

| Regression statistics | | < 2,000 m | 2,000 – 3,000 m | 3,000 – 4,000 m | > 4,000 m |
| --- | --- | --- | --- | --- | --- |
| Initial similarity | | 1 | 1 | 1 | 1 |
| n | | 2,850 | 1,225 | 231 | 120 |
| Linear  regression | a | -0.0005 | -0.00047 | -0.00052 | -0.00057 |
|  | b | 0.9843 | 0.9779 | 0.9506 | 0.9280 |
|  | *R^2^* | 0.750 | 0.634 | 0.639 | 0.567 |
|  | HD (km) | 969 | 1,017 | 867 | 751 |
|  | a | -0.1513 | -0.1123 | -0.1405 | -0.1450 |
| Logarithmic | b | 1.6581 | 1.4557 | 1.5627 | 1.5585 |
| regression | *R^2^* | 0.610 | 0.547 | 0.566 | 0.513 |
|  | HD (km) | 2,110 | 4,965 | 1,927 | 1,480 |
|  | a | -0.0007 | -0.0006 | -0.0007 | -0.0008 |
| Exponential | b | 1.023 | 0.9925 | 0.9757 | 0.9569 |
| regression | *R^2^* | 0.767 | 0.654 | 0.612 | 0.573 |
|  | HD (km) | 1,023 | 1,143 | 955 | 811 |

**Figure S2.** Relationship between the Simpson similarity of non-volant small mammals and geographic distance (km) for pairwise counties in the (a) < 2,000 m elevation zone (*n* = 2,850), (b) 2,000 – 3,000 m elevation zone (*n* = 1,225), (c) 3,000 – 4,000 m elevation zone (*n* = 231) and (d) > 4,000 m elevation zone (*n* = 120) of the Hengduan Mountains. For each elevation zone, the lines fitted indicate the linear, logarithmic and exponential regressions and all the regressions were significant at *P* < 0.001.


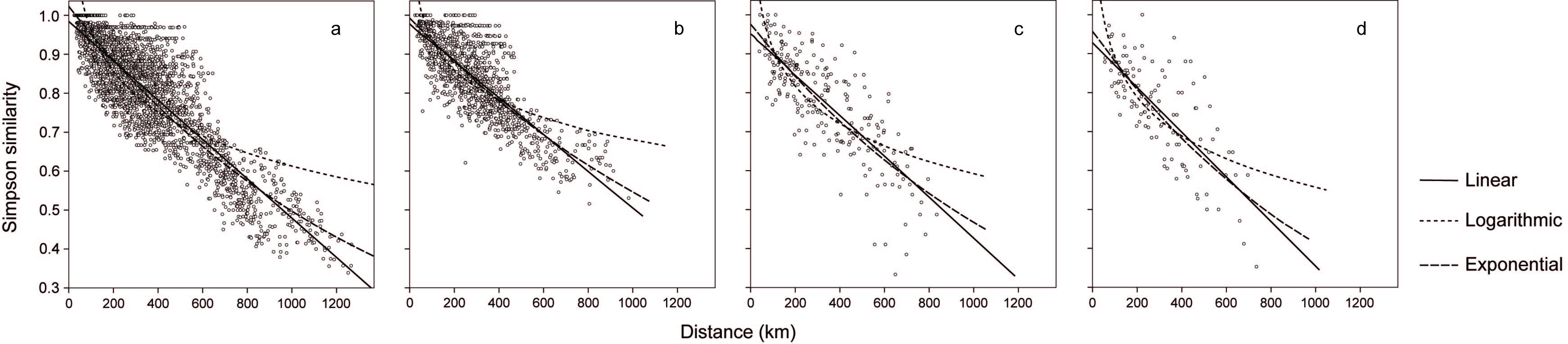

Supplement: Supplementary file 5 — Appendix S5. Comparison of the halving distances between Glires and insectivores in the entire area of the Hengduan Mountains (Table S1), the halving distance of each mammal group was calculated for the linear and logarithmic regression models between Jaccard similarity and geographic distance (km) (Figure S1); comparison of the halving distances of all non‐volant small mammal species among four elevation zones of the Hengduan Mountains (Table S2), the halving distance in each elevation zone was calculated for the linear, logarithmic and exponential regression models between Simpson similarity and geographic distance (km) (Figure S2). [file ECE3-6-0946-s005.doc]
