# Supplementary material for: Dispersal, niche, and isolation processes jointly explain species turnover patterns of nonvolant small mammals in a large mountainous region of China
Source: Ecol Evol. 2016 Jan 18;6(4):946–60. doi: 10.1002/ece3.1962 (PMC4761768; doi:10.1002/ece3.1962)
Supplement: Supplementary file 6 — Appendix S6. Results of the partial Mantel tests examining the correlations between the Jaccard similarity of non‐volant small mammals and the four explanatory factors in the entire area of the Hengduan Mountains (Table S1) and in the five longitudinal/latitudinal (21°–26°N: Table S2; 26°–30°N: Table S3; 30°–35°N: Table S4; 98°–102°E: Table S5; 102°–106°E: Table S6) zones of the region. [file ECE3-6-0946-s006.doc]

***Ecology and Evolution***

**Dispersal, niche and isolation processes** **jointly explain species turnover patterns of non-volant small mammals in a large mountainous region of China**

Zhixin Wen, Qing Quan, Yuanbao Du, Lin Xia, Deyan Ge and Qisen Yang*

*Corresponding author: Key Laboratory of Zoological Systematics and Evolution, Institute of Zoology, Chinese Academy of Sciences, 1 Beichen West Road, Beijing, 100101, China;

yangqs@ioz.ac.cn; telephone: +86-010-64807225

**Appendix S6**

**Results of the partial Mantel tests examining the correlations between the Jaccard similarity of non-volant small mammals and the four explanatory factors in the entire area of the Hengduan Mountains (Table S1) and in the five longitudinal/latitudinal (21° – 26°N: Table S2; 26° – 30°N: Table S3; 30° – 35°N: Table S4; 98° – 102°E: Table S5; 102° – 106°E: Table S6) zones of the region**

**Table S1.** Correlation coefficients of the partial Mantel tests examining the correlations between Jaccard similarity of non-volant small mammals and the four explanatory factors (geographic distance, environmental distance, difference in average elevation and difference in elevation range) in the entire area of the Hengduan Mountains. The *P*-value of each test was assessed based on 1,000 permutations with the significance (*P* < 0.05) shown by the bold character of the correlation coefficient.

|  | **Controlling the effect of** | | | |
| --- | --- | --- | --- | --- |
| **Explanatory Factor** | Geographic distance (km) | Environmental  distance | Difference in  average elevation (m) | Difference in elevation range (m) |
| Geographic distance (*R*) | NA | **-0.824** | **-0.796** | **-0.877** |
| Environmental distance (*R*) | **-0.275** | NA | **-0.378** | **-0.564** |
| Difference in average elevation (*R*) | **-0.442** | **-0.589** | NA | **-0.714** |
| Difference in elevation range (*R*) | **-0.156** | -0.026 | **-0.238** | NA |

NA: Not available

**Table S2.** Correlation coefficients of the partial Mantel tests examining the correlations between Jaccard similarity of non-volant small mammals and the four explanatory factors (geographic distance, environmental distance, difference in average elevation and difference in elevation range) in the 21° – 26°N zone of the Hengduan Mountains. The *P*-value of each test was assessed based on 1,000 permutations with the significance (*P* < 0.05) shown by the bold character of the correlation coefficient.

|  | **Controlling the effect of** | | | |
| --- | --- | --- | --- | --- |
| **Explanatory Factor** | Geographic distance (km) | Environmental  distance | Difference in  average elevation (m) | Difference in elevation range (m) |
| Geographic distance (*R*) | NA | **-0.665** | **-0.682** | **-0.737** |
| Environmental distance (*R*) | **-0.199** | NA | **-0.359** | **-0.439** |
| Difference in average elevation (*R*) | **-0.423** | **-0.473** | NA | **-0.557** |
| Difference in elevation range (*R*) | **-0.159** | -0.002 | **-0.201** | NA |

NA: Not available

**Table S3.** Correlation coefficients of the partial Mantel tests examining the correlations between Jaccard similarity of non-volant small mammals and the four explanatory factors (geographic distance, environmental distance, difference in average elevation and difference in elevation range) in the 26° – 30°N zone of the Hengduan Mountains. The *P*-value of each test was assessed based on 1,000 permutations with the significance (*P* < 0.05) shown by the bold character of the correlation coefficient.

|  | **Controlling the effect of** | | | |
| --- | --- | --- | --- | --- |
| **Explanatory Factor** | Geographic distance (km) | Environmental  distance | Difference in  average elevation (m) | Difference in elevation range (m) |
| Geographic distance (*R*) | NA | **-0.772** | **-0.767** | **-0.795** |
| Environmental distance (*R*) | -0.100 | NA | **-0.185** | **-0.315** |
| Difference in average elevation (*R*) | **-0.153** | **-0.252** | NA | **-0.361** |
| Difference in elevation range (*R*) | -0.137 | -0.139 | -0.153 | NA |

NA: Not available

**Table S4.** Correlation coefficients of the partial Mantel tests examining the correlations between Jaccard similarity of non-volant small mammals and the four explanatory factors (geographic distance, environmental distance, difference in average elevation and difference in elevation range) in the 30° – 35°N zone of the Hengduan Mountains. The *P*-value of each test was assessed based on 1,000 permutations with the significance (*P* < 0.05) shown by the bold character of the correlation coefficient.

|  | **Controlling the effect of** | | | |
| --- | --- | --- | --- | --- |
| **Explanatory Factor** | Geographic distance (km) | Environmental  distance | Difference in  average elevation (m) | Difference in elevation range (m) |
| Geographic distance (*R*) | NA | **-0.810** | **-0.829** | **-0.854** |
| Environmental distance (*R*) | **-0.371** | NA | **-0.438** | **-0.495** |
| Difference in average elevation (*R*) | -0.174 | -0.013 | NA | **-0.372** |
| Difference in elevation range (*R*) | **-0.316** | -0.036 | **-0.280** | NA |

NA: Not available

**Table S5.** Correlation coefficients of the partial Mantel tests examining the correlations between Jaccard similarity of non-volant small mammals and the four explanatory factors (geographic distance, environmental distance, difference in average elevation and difference in elevation range) in the 98° – 102°E zone of the Hengduan Mountains. The *P*-value of each test was assessed based on 1,000 permutations with the significance (*P* < 0.05) shown by the bold character of the correlation coefficient.

|  | **Controlling the effect of** | | | |
| --- | --- | --- | --- | --- |
| **Explanatory Factor** | Geographic distance (km) | Environmental  distance | Difference in  average elevation (m) | Difference in elevation range (m) |
| Geographic distance (*R*) | NA | **-0.855** | **-0.694** | **-0.911** |
| Environmental distance (*R*) | **-0.181** | NA | **-0.233** | **-0.608** |
| Difference in average elevation (*R*) | **-0.233** | **-0.721** | NA | **-0.825** |
| Difference in elevation range (*R*) | **-0.170** | -0.022 | 0.054 | NA |

NA: Not available

**Table S6.** Correlation coefficients of the partial Mantel tests examining the correlations between Jaccard similarity of non-volant small mammals and the four explanatory factors (geographic distance, environmental distance, difference in average elevation and difference in elevation range) in the 102° – 106°E zone of the Hengduan Mountains. The *P*-value of each test was assessed based on 1,000 permutations with the significance (*P* < 0.05) shown by the bold character of the correlation coefficient.

|  | **Controlling the effect of** | | | |
| --- | --- | --- | --- | --- |
| **Explanatory Factor** | Geographic distance (km) | Environmental  distance | Difference in  average elevation (m) | Difference in elevation range (m) |
| Geographic distance (*R*) | NA | **-0.864** | **-0.883** | **-0.896** |
| Environmental distance (*R*) | **-0.365** | NA | **-0.526** | **-0.535** |
| Difference in average elevation (*R*) | **-0.087** | **-0.227** | NA | **-0.465** |
| Difference in elevation range (*R*) | **-0.279** | **-0.159** | **-0.429** | NA |

NA: Not available
