# Supplementary material for: Dispersal, niche, and isolation processes jointly explain species turnover patterns of nonvolant small mammals in a large mountainous region of China
Source: Ecol Evol. 2016 Jan 18;6(4):946–60. doi: 10.1002/ece3.1962 (PMC4761768; doi:10.1002/ece3.1962)
Supplement: Supplementary file 7 — Appendix S7. Coefficients of determination (R 2) for the multiple regression analyses showing the contributions of the four explanatory matrices and the difference in area matrix to explaining the variation in the Jaccard similarity matrix of non‐volant small mammals in the entire area of the Hengduan Mountains and in the five longitudinal/latitudinal zones of the region. [file ECE3-6-0946-s007.doc]

***Ecology and Evolution***

**Dispersal, niche and isolation processes** **jointly explain species turnover patterns of non-volant small mammals in a large mountainous region of China**

Zhixin Wen, Qing Quan, Yuanbao Du, Lin Xia, Deyan Ge and Qisen Yang*

*Corresponding author: Key Laboratory of Zoological Systematics and Evolution, Institute of Zoology, Chinese Academy of Sciences, 1 Beichen West Road, Beijing, 100101, China;

yangqs@ioz.ac.cn; telephone: +86-010-64807225

**Appendix S7**

**Coefficients of determination (*R^2^*) for the multiple regression analyses showing the contributions of the four explanatory matrices and the difference in area matrix to explaining the variation in the Jaccard similarity matrix of** **non-volant small mammals in the entire area of the Hengduan Mountains and in the five longitudinal/latitudinal zones of the region**

**Table S1.** Coefficients of determination (*R^2^*) for the multiple regression analyses showing the contributions of the four explanatory matrices [geographic distance (GD), environmental distance (ED), difference in average elevation (DAE) and difference in elevation range (DER)] and the difference in area (DA) matrix to explaining the variation in the Jaccard similarity matrix of non-volant small mammals in the entire area of the Hengduan Mountains and in the five longitudinal/latitudinal zones of the region. The *R^2^* form the basis of the variation partitioning procedure described in “Materials and methods”, and each regression analysis was performed with 1,000 permutations to determine the statistical significance (*P-*values, all < 0.01).

| Explanatory matrices in the model | Entire  Hengduan Mountains | 21°–26°N zone | 26°–30°N zone | 30°–35°N zone | 98°–102°E zone | 102°–106°E zone |
| --- | --- | --- | --- | --- | --- | --- |
| (1) GD | 0.781 | 0.548 | 0.645 | 0.74 | 0.835 | 0.828 |
| (2) ED | 0.368 | 0.222 | 0.132 | 0.347 | 0.407 | 0.411 |
| (3) DAE | 0.518 | 0.307 | 0.158 | 0.192 | 0.699 | 0.228 |
| (4) DER | 0.073 | 0.036 | 0.055 | 0.136 | 0.06 | 0.196 |
| (5) GD+ED | 0.797 | 0.566 | 0.649 | 0.775 | 0.84 | 0.851 |
| (6) GD+DAE | 0.824 | 0.629 | 0.654 | 0.747 | 0.844 | 0.829 |
| (7) ED+DAE | 0.587 | 0.396 | 0.187 | 0.347 | 0.715 | 0.442 |
| (8) GD+ED+DAE | 0.829 | 0.636 | 0.654 | 0.778 | 0.847 | 0.851 |
| (9) GD+ED+DAE+DER  (10)GD+ED+DAE+DER+DA | 0.831  0.837 | 0.643  0.645 | 0.658  0.693 | 0.781  0.782 | 0.848  0.85 | 0.854  0.855 |
